# Retrospective Analysis of the Serologic Response to the Treatment of Syphilis During Pregnancy

**DOI:** 10.1155/S1064744997000070

**Published:** 1997

**Authors:** Henry L. Galan, Juan F. Montalvo, John Deaver

**Affiliations:** 1 Department of Obstetrics, Gynecology, and Reproductive Sciences University of Texas Health Science Center Houston TX USA; 2 Department of OB/GYN University of Colorado Health Science Center Campus Box B-198 4200 E. 9th Avenue Denver CO 80262 USA

## Abstract

*Objective:* The purpose of this study was to assess the effect of several maternal variables on the serologic response following the treatment of syphilis in pregnancy.

*Methods:* A 5-year chart review identified 95 patients coded with syphilis at Hermann Hospital. Inclusion criteria were 1) serologically confirmed syphilis infection during the index pregnancy, 2) complete treatment during the index pregnancy, and 3) minimum of one follow-up rapid plasma reagin (RPR) titer. Forty-nine of 95 patients met the inclusion criteria. Treatment response was evaluated by comparing each post-treatment titer of a patient to her pretreatment titer. Each comparison was considered an “observation.” Each observation was classified as either a positive response (≥4-fold titer decline) or a negative response (<4-fold titer decline). Maternal variables assessed included 1) prior history of syphilis untreated or incompletely treated prior to the index pregnancy, 2) gestational age, 3) titer level, 4) unknown duration, 5) positive response at 1 month, 6) positive response at 2 months, 7) positive response at >3 months, and 8) race.

*Results:* A positive response following treatment was significantly more likely if there was no prior history of syphilis or if there was a high initial RPR titer (>32). Only 33/54 (61%) observations at or greater than 3 months had a positive response.

*Conclusions:* Our study suggests that an absence of a history of syphilis and an initial high RPR titer are predictive of a positive response following appropriate treatment. Given the low percentage of observations with a positive response at 3 months, we speculate that we may be undertreating our pregnant patients with syphilis infection.


Infectious Diseases in Obstetrics and Gynecology 5:23–28 (1997)
© 1997 Wiley-Liss, Inc.

### Retrospective Analysis of the Serologic Response to the Treatment of Syphilis During Pregnancy

**Henry L. Galan,* Juan F. Montalvo, and John Deaver**
*Department of Obstetrics, Gynecology, and Reproductive Sciences, University of Texas Health Science Center, Houston, TX*

### Abstract

*Objective:* The purpose of this study was to assess the effect of several maternal variables on the serologic response following the treatment of syphilis in pregnancy.

*Methods:* A 5-year chart review identified 95 patients coded with syphilis at Hermann Hospital. Inclusion criteria were 1) serologically confirmed syphilis infection during the index pregnancy, 2) complete treatment during the index pregnancy, and 3) minimum of one follow-up rapid plasma reagin (RPR) titer. Forty-nine of 95 patients met the inclusion criteria. Treatment response was evaluated by comparing each post-treatment titer of a patient to her pretreatment titer. Each comparison was considered an "observation." Each observation was classified as either a positive response (≥4-fold titer decline) or a negative response (<4-fold titer decline). Maternal variables assessed included 1) prior history of syphilis untreated or incompletely treated prior to the index pregnancy, 2) gestational age, 3) titer level, 4) unknown duration, 5) positive response at 1 month, 6) positive response at 2 months, 7) positive response at >3 months, and 8) race.

*Results:* A positive response following treatment was significantly more likely if there was no prior history of syphilis or if there was a high initial RPR titer (>32). Only 33/54 (61%) observations at or greater than 3 months had a positive response.

*Conclusions:* Our study suggests that an absence of a history of syphilis and an initial high RPR titer are predictive of a positive response following appropriate treatment. Given the low percentage of observations with a positive response at 3 months, we speculate that we may be undertreating our pregnant patients with syphilis infection. Infect. Dis. Obstet. Gynecol. 5:23–28, 1997.
© 1997 Wiley-Liss, Inc.

Key words
rapid plasma reagin; venereal disease research laboratory test; syphilotherapy; serology; titers

Syphilis has sustained a recent resurgence in the United States. The incidence of syphilis in 1990 is the highest since 1950.[1] Additionally, the incidence of syphilis in pregnancy and of congenital syphilis has risen in parallel fashion.[1] According to the Centers for Disease Control (CDC), 35% of neonatal infections are related to treatment failures which can occur as a result of reinfection, severe infection, or treatment late in pregnancy.[2,3] Currently, the recommended treatment for syphilis in pregnancy is based on guidelines set by the CDC which are based on experience with nonpregnant patients. Several studies have addressed the ineffectiveness of treatment of syphilis during pregnancy.[4–6]

In 1993, Nathan et al.[6] demonstrated that a single dose of 2.4 million units of benzathine penicillin G given at term results in a wide range of penicillin levels in various maternal-fetal compartments. They speculated that altered pharmacokinetics may affect the efficacy of this drug for prevention of congenital syphilis in the near term ges-

*Correspondence to: Dr. Henry L. Galan, Department of OB/GYN, University of Colorado Health Science Center, Campus Box B-198, 4200 E. 9th Avenue, Denver, CO 80262. E-mail: galanh@jove.uchsc.edu

Received 22 August 1996
Accepted 5 May 1997

TABLE 1. CDC guidelines for treatment of syphilis

| Stage of syphilis | Treatment |
|---|---|
| Early syphilis (1°, 2°, latent syphilis of <1 year duration) | Benzathine penicillin G 2.4 MU IM × 1 |
| Latent syphilis of unknown duration or >2 years duration, cardiovascular, or late benign syphilis | Benzathine penicillin G 2.4 MU IM weekly × 3 weeks |
| Neurosyphilis | Aqueous crystalline penicillin G 2–4 MU IV every 4 h for 10 days, *or* aqueous procaine penicillin G 2.4 MU IM plus probenecid 500 mg PO 4 times daily; both daily for 10 days to be followed by benzathine penicillin G 2.4 MU IM weekly for 3 weeks |

tation. Pregnancy-related physiologic changes such as expanded blood volume and volume of distribution is one of several variables that could potentially affect nonspecific serologic markers of syphilis such as the venereal disease research laboratory (VDRL) or rapid plasma reagin (RPR) tests. Adequate treatment of syphilis is conventionally documented in nonpregnant individuals by a 4-fold decrease in VDRL or RPR titers over a 3-month period of time.[7–11] While the expected decrease in RPR is based on studies of nonpregnant patients, this expected decrease is also used in the pregnant patient to assess adequacy of treatment or the need for retreatment. No study has specifically evaluated the pattern of post-syphilis treatment nontreponemal antibody titers in pregnancy. The purpose of this study is to assess the effect of several maternal variables on the serologic response in patients treated for syphilis during pregnancy.

### SUBJECTS AND METHODS

This is a retrospective study of pregnant patients who were evaluated for syphilis over a 5-year period (1988–1993) at the University of Texas Health Science Center at Houston and Hermann Hospital. Administrative approval was obtained from the Committee for Protection of Human Subjects for a chart review. Patient inclusion criteria were 1) serologic documentation of syphilis infection during the index pregnancy, 2) complete treatment given during the index pregnancy, and 3) completion of treatment using CDC guidelines. Exclusion criteria were 1) a history of syphilis completely treated prior to the index pregnancy, 2) a treatment regimen outside of the CDC guidelines, 3) lack of any follow-up titers, 4) completion of treatment in the postpartum period, and 5) human immunodeficiency virus (HIV) positivity.

The diagnosis of syphilis was based on serologic testing using the RPR and confirmed with the microhemagglutination test for *T. pallidum* (MHA-TP). Either a 4-fold increase in titer or a titer of ≥1:16 in a patient with a previous history of syphilis treatment would be considered inadequate treatment or reinfection. Identification of spirochetes from primary or secondary lesions by darkfield microscopy was also considered diagnostic. Treatment of syphilis was in accordance with CDC guidelines which are listed in Table 1. RPR titers were used to evaluate the response to treatment. At our institution, RPR titers are performed monthly for the remainder of the pregnancy following completion of syphilis treatment. Staging was documented according to the stage at the time of treatment as recorded in the chart. Treatment was documented either by contact with the sexually transmitted disease (STD) Hotline or based on what was documented in the chart. The patient's partner is typically treated and is encouraged to follow-up at the local health department, however, partner compliance was not assessed in this study.

Maternal variables assessed included 1) history of syphilis either untreated or incompletely treated prior to the index pregnancy (completely treated pregestational syphilis patients were excluded), 2) stage of disease as assigned at the time of treatment, 3) gestational age at the time of treatment, 4) pretreatment RPR titers, 5) post-treatment RPR titers, and 6) race. These data were presented in dichotomous fashion for purposes of analysis. Therefore, the maternal variables for each patient were categorized as follows: 1) history vs. no history of pregestational syphilis, 2) unknown vs. known duration of syphilis, 3) early (<20 weeks gestation) vs. late gestational disease, 4) high (≥1:32) vs. low initial titer, 5) post-treatment titer at 1 month (±2

weeks) vs. after 1 month, 6) post-treatment titer at 2 months (±2 weeks) vs. after 2 months, 7) post-treatment titer at 3 months (±2 weeks) vs. after 3 months, and 8) black vs. non-black race. High or low pretreatment titer levels were divided into ≥1:32 or <1:32, respectively. This number was chosen because 1:32 was the median titer of all titers recorded.

Treatment response was assessed by comparing each patient's post-treatment titer to her pretreatment titer. Each comparison was considered an "observation." For example, if a patient had only a pretreatment titer available, this would be "0" observations, and this patient would be excluded. If a patient had a pretreatment titer and one post-treatment titer, this would be considered one observation. Two post-treatment titers would be considered two observations, and in this case the comparison of the pretreatment titer to the first post-treatment titer would be one observation, and the pretreatment titer to the second post-treatment titer would be the second observation. Observations were not made between post-treatment titers, but only between pretreatment and post-treatment titers. Each observation was categorized as either a positive response (≥4-fold titer decline) or negative response (<4-fold titer decline) to treatment. In our laboratory, RPR titers are reported as tube dilutions. A 2-tube dilution is equivalent to a 4-fold decline in titers (i.e., 1:16 to 1:4 response is a 2-tube dilution decline or a 4-fold decline).

### Statistical Analysis

Statistical analysis was performed with the True Epistat statistical package (Epistat Services, Richardson, TX). Maternal variables and their impact on positive titer responses were analyzed using Fisher's exact or chi-square tests and $P < 0.05$ was considered significant. For assessing the effect of race on positive titer response, the contingency table was subdivided and analyzed with Fisher's exact test. Because the contingency table for race was subdivided, the Bonferroni inequality was applied and the $P$ value adjusted from <0.05 to <0.02.

### RESULTS

A computer-generated report of pregnant patients with a code of syphilis between January 1, 1988, and December 31, 1993, revealed a total of 290 patients. Of these 290 patients, 95 were followed in the University of Texas Women's Clinic and had complete records. Of these 95, 41 had a history of appropriately treated syphilis prior to the index pregnancy, and were therefore excluded. An additional 5 patients were excluded because only the pretreatment titer was available. Of the 95 patients, a total of 49 met the inclusion criteria for the study. These 49 patients provided a total of 98 "observations." Table 2 shows the demographic data of the study population. In addition, 36 were without a history of previous syphilis infection. Two patients had a negative RPR screen on their initial prenatal visit with a subsequent positive RPR at 28 weeks. The majority of patients (>95%) had an initial RPR screen that was positive. The majority of patients had early latent syphilis (24) and syphilis of unknown duration (20). Two of the 49 patients were HIV positive and were excluded because of reports that RPR titers may fall slower in patients who have HIV infection. CDC treatment guidelines were followed and there were no penicillin allergic patients.

**TABLE 2. Demographic data of study population**

| | |
|---|---|
| Total study patients | 49 |
| Maternal age, years (range) | 22.8 (15–40) |
| EGA at initial prenatal visit, weeks (range) | 24.9 (9–40) |
| Parity | |
| Nulliparous | 11 (22%) |
| Multiparous | 38 (78%) |
| Race | |
| Black | 44 (90%) |
| Hispanic | 4 (8%) |
| White | 1 (2%) |
| Stages of syphilis | |
| Primary | 3 |
| Secondary | 2 |
| Early latent | 24 |
| Unknown duration | 20 |

Table 3 shows the effect of maternal variables on positive responses. Two of the 8 variables significantly impacted positive titer responses. Those patients with a pregestational history of syphilis (13/32) were significantly less likely ($P < 0.05$) to have a positive response than those without a previous history of syphilis (41/66). Those patients with a high RPR titer (36/51) were significantly

TABLE 3. Maternal variables having a positive response (≥4-fold decrease in titer) with corresponding $P$ values

| Maternal variables | Positive treatment responses |
|---|---|
| Prior history | |
| Yes | 13/32 (40%)* |
| No | 41/66 (62%)* |
| Early disease | |
| Yes | 21/49 (43%) |
| No | 17/49 (35%) |
| Initial RPR | |
| High (≥1:32) | 36/51 (70%)* |
| Low (<1:32) | 18/47 (30%)* |
| Duration | |
| Known | 33/64 (51%) |
| Unknown | 21/34 (62%) |
| Black race | |
| Yes | 52/88 (59%) |
| No | 4/10 (40%) |
| Observations | |
| At 1 month | 10/23 (43%) |
| vs.>1 month | 44/75 (59%) |
| At 2 months | 9/21 (43%) |
| vs.>2 months | 34/54 (63%) |
| At 3 months | 11/20 (55%) |
| vs.>3 months | 22/34 (65%) |

*$P < 0.05$.

more likely ($P < 0.005$) to have a positive response than those with low titers (18/47). The other 6 variables were found not to be significant factors for a positive response. The number of observations showing a positive treatment response when the post-treatment titers were obtained at 1 month (1 month ± 2 weeks) was not significantly different when compared to those in which the post-treatment titer was obtained at >1 month (i.e., 2 months, 3 months, etc.). The same nonsignificant findings were encountered for the observations of post-treatment titers at 2 months (2 months ± 2 weeks) when compared to those in which the post-treatment titers were obtained at >2 months (i.e., 3 months, 4 months, etc.). The same findings were also noted for the observations of post-treatment titers at 3 months (3 months ± 2 weeks) when compared to those in which the post-treatment titers were obtained at >3 months.

### DISCUSSION

A number of studies suggest that certain infections have a higher incidence in pregnancy and that they may be more difficult to treat. These include poliomyelitis, hepatitis A and B, and malaria.[12–15] Syphilis may also be considered in that group. The reason for the difficulty of syphilis treatment in pregnancy remains to be elucidated, but may be due to an altered immune response (acceptance of a partially allogenic fetus), reinfection, severity of infection, or that the method of assessment of cure is altered by physiologic volume changes in pregnancy. While the treatment of syphilis in the nonpregnant patient is very effective, treatment in the pregnant patient may have a high failure rate accounting for as much as 35% of neonatal infections.[2,3] Our findings show that only 55% of observations where the post-treatment titer was obtained at 3 months demonstrated a positive response (4-fold titer decline), and only 65% of observations in which the post-treatment titer was obtained at >3 months demonstrated a positive response. This suggests inadequate treatment which may contribute to the relatively high failure rate seen in pregnancy. As described by Nathan et al.,[6] the causes are likely to be multifactorial and may include advanced fetal disease, the Jarisch-Herxheimer reaction, altered penicillin pharmacokinetics in pregnancy, and altered maternal and fetal immune responses.

Nathan et al.[6] speculated that physiological changes occurring in pregnancy may result in alterations of penicillin pharmacokinetics leading to reduced penicillin levels. Changes such as expanded blood volume and volume of distribution could also potentially affect the way that nonspecific serologic markers of syphilis such as the VDRL or RPR respond in pregnancy. This is a critical issue considering that the assessment of successful treatment or the need for retreatment is based on convalescence of VDRL or RPR titers. If the titer levels and patterns differ from the nonpregnant state, then perhaps the normally expected 4-fold decrease over a 3-month period of time that is seen in the nonpregnant state should be reconsidered for the pregnant female. Depending on the rapidity or delay of titer decline, we may be over- or undertreating our patients. Our study suggests that of the maternal variables studied, an absence of a pregestational history of syphilis and a high initial titer are significant factors for predicting a positive response. Our finding that a pregestational history of syphilis alters the rate of RPR titer decline in the subsequent pregnancy in comparison with those patients without a history of syphilis may be due to previous inadequate treatment or persistent infection which over time had become more advanced. This is consistent with a recent abstract by McFerlin et al. (IDSOG, 1995) which demonstrated that women who have had syphilis in a previous pregnancy are at high risk of delivering an infant with congenital syphilis in the subsequent pregnancy.

Ideally, a study assessing the response of syphilotherapy in pregnancy should be performed in a prospective controlled fashion using matched, nonpregnant patients as controls. Our study was limited by the small number of patients eligible for the study which required us to establish "observations" to assess positive treatment responses to therapy. Positive treatment responses should ideally be assessed within each individual patient instead of as "observations" which combine the data from all patients. There is sufficient variation in laboratory serologic work such that a failure to reach a 4-fold decrease after 3 months may not necessarily indicate inadequate treatment since the next monthly titer may subsequently reflect an appropriate decline in titer. A second limitation is from an analytical viewpoint where the maternal variables should be treated as independent variables, thereby implying the need for a multivariate analysis. A third potential retrospective bias is that our data were obtained by chart review and we were dependent on accurate documentation of staging and treatment. This could result in misclassification of patients, e.g., including patients into the study with low titers who were previously treated which would bias "low titer" patients as nonresponders. However, the inclusion criteria for "low titer" patients were stringent and required that patients meet the CDC recommendations for infection of a 4-fold rise in RPR titer. A fourth limitation is the lack of available neonatal follow-up which is needed to substantiate speculations regarding adequacy of maternal and fetal therapy.

Another finding in our study which could potentially have a clinical impact was the lack of a significant difference in positive responses when observations in which post-treatment titers at 1 month were compared to observations in which post-treatment titers were obtained at >1 month. This suggests that we may be able to detect early on whether a patient is going to respond appropriately to treatment. However, this interesting finding should be tempered by the small number of patients in this study. While our study has some limitations, it calls attention to the need for further studies regarding the proper interpretation of serologic titers in pregnancy which may be different from the nonpregnant patient. Assuming that titers fall the same in the pregnant and nonpregnant state and that the CDC guidelines for assessing appropriate titer decline to therapy are applicable in pregnancy, our data suggest that we are undertreating our pregnant patients with syphilis infections or that patients are becoming reinfected during pregnancy either by an untreated partner or inadequate fetal treatment. This is consistent with other studies in the literature. While the endpoints of several studies addressing the adequacy of current CDC treatment guidelines have included congenital syphilis and/or maternal-fetal penicillin levels, our study is the first to address the endpoint with respect to maternal serology and can serve as a pilot study on which future studies may be based. In addition, further studies are needed to compare the serologic responses between the pregnant and nonpregnant patient.

### REFERENCES

1. Centers for Disease Control: Syphilis and congenital syphilis, United States, 1985–1988. MMWR 37:486–489, 1988.
2. Centers for Disease Control: Congenital syphilis, United States, 1983–1985. MMWR 35:625–628, 1986.
3. Centers for Disease Control: Guidelines for the prevention and control of congenital syphilis. MMWR 37:s-1, 1988.
4. Mascola L, Pelosi R, Alexander CD: Inadequate treatment of syphilis in pregnancy. Am J Obstet Gynecol 150:945–947, 1984.
5. Zenker PN, Berman SM: Congenital syphilis: Trends and recommendations for evaluation and management. Pediatr Infect Dis 10:516–522, 1991.
6. Nathan L, Bawdon RE, Sidawi E, Stettler RW, McIntire DM, Wendel GD: Penicillin levels following the administration of benzathine penicillin G in pregnancy. Obstet Gynecol 82:338–342, 1993.
7. Brown ST, Zaidi A, Larsen SA, Reynolds GH: Serological response to syphilis treatment—A new analysis of old data. JAMA 253:1296–1299, 1985.
8. Magnuson HJ, Thomas EW, Olansky S, et al.: Inoculation of syphilis in human volunteers. Medicine 35:33–82, 1956.
9. Schroeter AL, Lucas JB, Price EV, et al.: Treatment for early syphilis and reactivity of serologic tests. JAMA 221:471–476, 1972.

10. Creasy RK, Resnik R: Preterm labor and delivery. In: Maternal-Fetal Medicine: Principles and Practice. Philadelphia: W.B. Saunders, pp 494–520, 1994.
11. Sweet RL, Gibbs RS: Sexually transmitted diseases. In: Infectious Diseases of the Female Genital Tract. 2nd ed. Baltimore: Williams & Wilkins, pp 133–181, 1990.
12. Siegel M, Greenberg M: Incidence of poliomyelitis in pregnancy. N Engl J Med 253:841–853, 1955.
13. Borhanmanesh F, Haghighi P, Hekmat K, et al.: Viral hepatitis during pregnancy. Gastroenterology 64:304–309, 1973.
14. Morrow RH, Smetana HF, Sai FT, et al.: Unusual features of viral hepatitis in Accra, Ghana. Ann Intern Med 68:1250–258, 1968.
15. Diro M, Beyboun SN: Malaria in pregnancy. South Med J 75:959–966, 1982.
16. McFerlin BL, Bottoms SF: Syphilis during pregnancy: the next pregnancy. Annual Meeting Infectious Diseases Society for Obstetrics and Gynecology, Grand Travers, MI, August 2–5, 1995.
